# Public perception of cultural ecosystem services in historic districts based on biterm topic model

**DOI:** 10.1038/s41598-024-62770-0

**Published:** 2024-05-22

**Authors:** Ying Pan, Nik Hazwani Nik Hashim, Hong Ching Goh

**Affiliations:** Centre for Sustainable Urban Planning and Real Estate (SUPRE), Faculty of Built Environment, Universiti Malaya, 50603 Kuala Lumpur, Malaysia

**Keywords:** Biterm topic model, Public perception, Importance-performance analysis, Historic districts, Satisfaction analysis, Sentiment analysis, Cultural ecosystem services (CES), Importance analysis, Ecosystem services, Environmental social sciences

## Abstract

Historic districts are integral components of urban space, possessing diverse ecosystems that can offer various cultural services to the public. Urbanization and tourism development have led to the degradation of the ecological landscapes within historic districts, impacting sustainable development. Incorporating Cultural Ecosystem Services (CES) into the environmental research of historic districts can meet people's spiritual needs, enhance intangible benefits for humanity, and promote the conservation of the ecological environment within historic districts. Therefore, this study conducted perceptual quantification research on CES in four typical historic districts in Fuzhou City, crawling the online comment data through Python, mined its potential themes using Biterm Topic Model (BTM), and extracted and categorized the indicators of CES of historic districts by combining with expert consultation; meanwhile, the satisfaction of CES of historic districts is further explored with the help of two methods, namely, sentiment analysis and Importance-Performance analysis (IPA), and summarized the public perception of CES of historic districts. The results of the study show that: (1) the dimensions of public perception of CES in urban historic districts include Cultural Heritage, Leisure Tourism, Aesthetic Enjoyment, Spiritual Fulfillment, Inspiration, and Science Education six indicators, of which Leisure Tourism is most easily perceived by the public, but its satisfaction is not high; (2) the public's perception of positive emotions towards the CES of historic districts in Fuzhou is greater than negative emotions, with positive emotions accounting for 80.61%; (3) the public's overall satisfaction with the CES of Fuzhou's historic districts is high, and according to the final analysis results of the IPA, the four historic districts of Fuzhou are respectively proposed to improve the opinions. Based on big data, this study explores the public perception characteristics of CES in Fuzhou historic districts to promote its sustainable development and improve public well-being, which is of great significance to protecting the ecological environment of historic districts and improving the quality of cultural services.

## Introduction

### Research background

Urbanization has brought greater pressure to urban ecology^1^. The expansion of cities and the increase in population not only lead to environmental degradation but also have a significant impact on the overall appearance of historic districts. The spatial form of historic districts has experienced a situation of "loss of ecological landscapes and deconstruction of green systems,"^2^ affecting sustainable development. Historic districts, bearing witness to urban development, have become an important aspect of urban diversity and sustainable development due to their dual attributes of cultural heritage and community life^3^. Due to conflicts between economic development, cultural heritage protection, and sustainable development, people are calling for a new way of thinking and methods to rebuild ecological, safe, and comfortable historical and cultural districts^4^.

At the same time, with the development of the economy and the improvement of living standards, the main contradictions in Chinese society have changed, and people's desire to pursue the needs of a better life has become stronger^5^. The need for a better life not only refers to meeting the public’s material needs but also meeting the public’s needs in culture, ecology, education, sports, etc. Vigorously improve service quality and service efficiency to achieve the all-round development of people. The proposal of Cultural Ecosystem Services (hereinafter referred to as CES) focuses on emphasizing its important value to the human spiritual level. Its connotation is to explore how to meet human spiritual needs and increase human non-material benefits, thereby improving people's well-being^6^. Historic districts also have diverse types of ecosystems, capable of providing various CES to the public. In the future, they will become the core for delivering economic benefits and social welfare^7^.

### Literature review

Historic districts refer to regions of a certain scale, with many preserved historical relics and well-protected historical features^8^. The disciplines used to study them vary, from conservative viewpoints focusing on the protection of cultural heritage to ecological and cultural resilience and cultural evolution, history Neighborhoods have received the attention of many scholars. Although most of the current research on historic districts by scholars focuses on the protection^9^, regeneration^10^, vitality improvement^11,12^, and tourism experience^13,14^ of historic districts. But in fact, some scholars have used "ecology" as a keyword to study historic districts. For example, in 2016, Shen Suyan and Ai Lijun proposed that "if there is no coordination between the development of the block and the ecological environment, it will lead to the destruction of the historic district and the deterioration of the living environment of residents, laying hidden dangers for its sustainable development."^15^; In 2017, Chen Shuo, Jiang Bin and Song Shoujian proposed "to interpret the design of environmental sketches in historical and cultural districts based on the perspective of ecological design and to rethink the relationship between environmental sketches and ecological design"^16^; Hu Changjuan and Gong Cong conducted a comparative analysis the revitalization of four typical historic districts explores the creation of ecological historic districts^2^; in 2019, Wu Lingrong and Xiao Rong proposed a study on the regeneration of historic districts based on ecosystem thinking^17^. However, there is still a gap in researching historical districts from the perspective of CES. With the development of the economy and the improvement of people's living standards, the cultural service functions of historic districts will attract more and more people's attention.

The Millennium Ecosystem Assessment (MEA, 2005) defines ecosystem services (hereinafter referred to as ES) as "the benefits that people obtain from ecosystems", which includes four interrelated categories: Supporting Services, Provisioning Services, Regulating Services and Cultural Services. This comprehensive tool promotes sustainable development and comprehensive assessment of the natural and cultural values of landscapes, with a focus on human well-being and guidance for decision-making^18^. CES is an important component of ES. Its connotation is the non-material benefits that humans obtain from the ecosystem, including aesthetic experience, spiritual satisfaction, thinking, entertainment and cognitive ability development, etc. Compared with the other three service types, cultural services are directly experienced and intuitively appreciated by humans. Ecosystems with higher cultural service functions often help to increase public support for protecting ecosystems. At the same time, CES cannot be replaced if it degrades^19^. In cities, the value of CES is huge, especially the importance of leisure and well-being that may exceed other ecosystem services^20^ because CES is easier to experience, understand, and appreciate than other ecosystem service types^21^. The objects of quantitative research on CES are more concentrated in rural areas^22,23^, mountains^24,25^, river landscapes^26^, wetland parks^27^, etc., and limited attention has been paid to urban CES, especially considering the benefits that the public can obtain from it^28^. One of the reasons is that urban ecosystems are highly complex. Secondly, because CES is "intangible" and "immaterial" compared with other services, the lack of qualitative and quantitative data is a major obstacle to quantitative research on it^29,30^. Currently, the research focus on CES also centers on quantification, with the main quantification methods being monetization, model estimation, and public participation mapping^31^. Among them, monetization quantifies the value of CES by using market valuation or willingness-to-pay valuation. Such methods mostly rely on questionnaire surveys, which are more subjective and have a limited number of samples^32^; the model estimation method is based on geographical information and uses models. When assigning values to regional CES, this method tends to ignore the perceptual characteristics of CES^33^; public participation in mapping combines human perception with geographical space in the form of a questionnaire. Due to the complexity of the operation, there is also a limitation of limited sample size^34^. In recent years, with the in-depth development of the Internet, a large number of scenic spot reviews have appeared on major websites, and these online text data have become new materials for studying perceptual characteristics. Compared with traditional questionnaires, online text is easy to collect and the amount of data is huge, providing new ideas for quantitative research on CES.

Although historic districts are not the primary source of CES in urban ecosystems, prioritizing the landscape spaces within historic districts can promote their sustainable development and enhance human well-being. This will encourage public awareness and attention to the surrounding environmental ecosystems.

### Research methods and purposes

With the development of the Internet, more and more people like to hit tourist spots on online platforms and leave their comments to record their trips. Compared with the long-winded travelogues in the past, most of these comments are in the form of short textbooks, potentially including only a few words. With the popularity of large amounts of short text review data, it is very meaningful to analyze it and understand some phenomena from it^35^. Currently, analyzing the content of short texts is mainly applied in the fields of visitor preference^36^, satisfaction^37^, sentiment analysis^38^, etc. It is also possible to infer their potential themes by studying the short text, such as theme model characterization^39,40^, user interest analysis^41^, and emerging theme detection^42^. Historic districts, as relatively popular tourist attractions in China, can be retrieved from a large number of comments on major online platforms, and the comments data can reflect the main perceptions and emotional attitudes of tourists towards specific historic districts^43^, That's why social media is becoming an important way to get audience perception data^44^. Compared with traditional questionnaires and interviews, these review data have the advantages of large sample size and easy collection, which better reflect the public's perceptions^45^, provide a new way of data collection for quantitative research on CES, and contribute to the diversification of data sources and improve the credibility of the research results^46^.

With the rise of machine learning, more and more people are using natural language processing techniques to analyze and process collected web data^47^. Among them, the topic model can effectively mine the topic information implied by the words in the text, and there are two common topic models: the latent Dirichlet allocation (LDA) and the biterm topic model (BTM)^35,48^. Compared with the LDA, the recently developed BTM efficiently models short texts by capturing rich global word co-occurrence information, which is more suitable for short text topic extraction as well as helpful for short text clustering^49^and topic learning^50^.So the article introduces the BTM online algorithm to process large-scale network short text data, providing technical support for CES perception research and exploring the public's perception characteristics of CES in historic districts.

This article hopes to call on everyone to pay attention to the ecological environment and cultural value of the neighborhood through the study of CES in historic districts, which plays an important role in the sustainable development of historic districts and even cities. Therefore, this study chooses to carry out quantitative research on CES in urban historic districts.

## Research methods

### Study region

**Figure 1:**
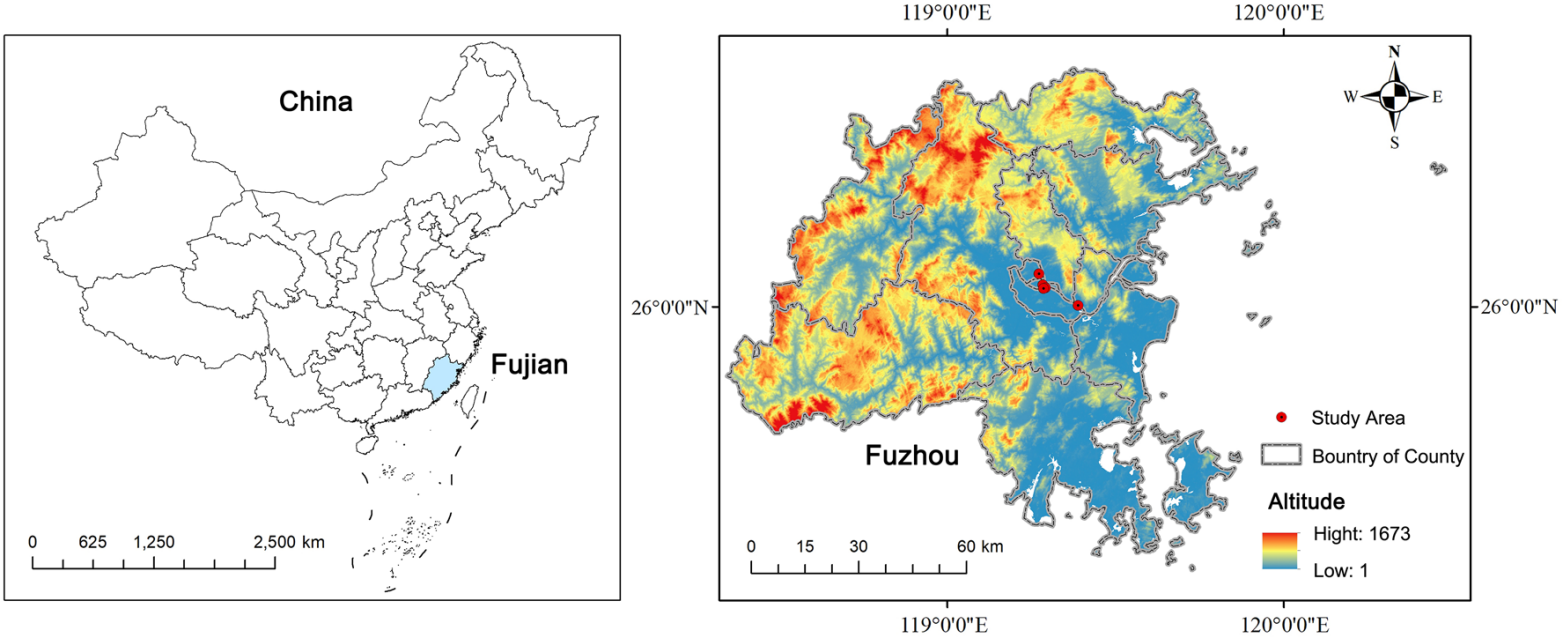
Study area and elevation map.

**Figure 2:**
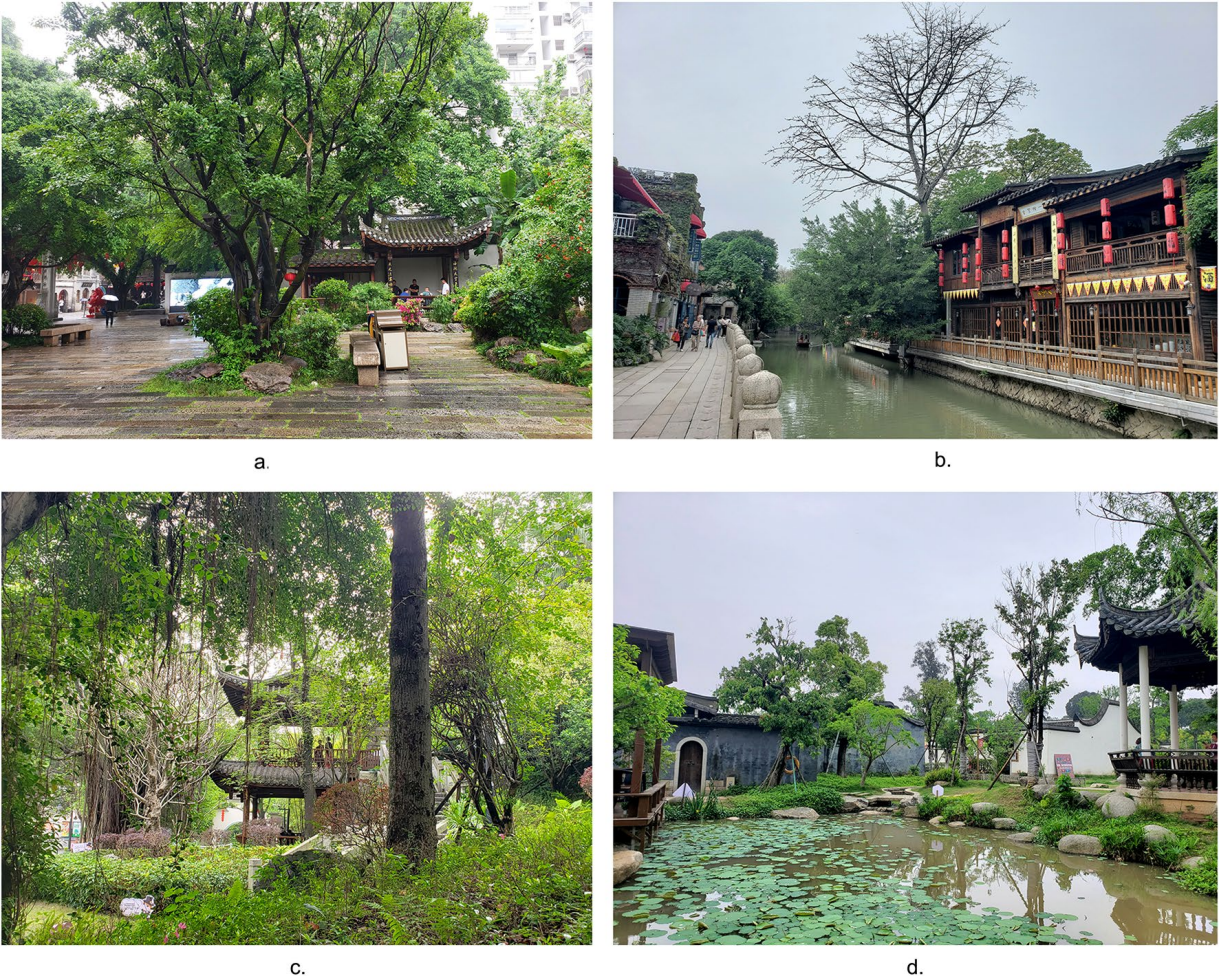
Photos of four historic districts.

Located in the central region of Fujian Province, Fuzhou city is also the capital of Fujian Province and the center city of the West Coast Economic Zone, with more than 7,000 years of history and culture and more than 2,200 years of history of founding the city. The specific study area and elevation map are shown in Fig. 1. In 1986, Fuzhou was announced by the State Council as the second batch of national historical and cultural cities. The long history of Fuzhou has accumulated a deep cultural heritage, leaving behind numerous historical and cultural preservation areas, cultural relics and monuments, historical and cultural resources are very rich. In recent years, Fuzhou's tourism market has been extremely active, receiving 78,615,600 tourists in 2022 year, and the city government is also trying its best to create cultural tourism areas to activate the cultural tourism market. Currently, Fuzhou has renewed four well-known historic districts, the on-site photos are shown in Fig. 2, namely Three Lanes and Seven Alleys historic district (Fig. 2a), Shangxiahang historic district (Fig. 2b), Yantai Hill historic district (Fig. 2c), and Liangcuo historic district (Fig. 2d). As the main gathering place of different cultures in Fuzhou, these four historic districts have a variety of cultural genres and can provide visitors with rich and diverse cultural services.

### Data collection and pre-processing

The data for this study comes from user comment data from www.dianping.com, which was launched in 2003 and is the earliest consumer review website in China. As of April 2023, Dazhong Dianping had 121 million monthly active users, and has been committed to communicating and aggregating urban consumer experiences, and has a high advantage in terms of the fairness of reviews. It is also the site that records the highest number of reviews about historic district. By crawling the comment data in this website through Python, the number of comments crawled as of December 13, 2023, totaled 15,055, of which Three Lanes and Seven Alleys, as a popular 5A scenic spot in Fuzhou, has the most number of comments, amounting to 8,239; Liangcuo, as a newly developed historic district, has the fewest comments. To ensure the high quality of the data, preliminary processing of the data is carried out, which includes: (1) deletion of meaningless short fields, such as "for", "came", "clocked in" and so on, which are less than 5 characters; (2) deleting particularly long and repetitive comments, as well as comments that belong to copy and paste; (3) deleting comments that are blank or only have punctuation marks; (4) translating comments that use different languages, such as Japanese, English, etc. The final comment data is 14,681.

At the same time, the final comment data are preprocessed by the JIEBA Chinese text processing function, and the preprocessing content includes: (1) adding processing of proper nouns, such as the characteristic attractions "Love Tree", "Santong Bridge" "Three Lanes and Seven Alleys", "Yantai Hill", etc., to avoid algorithmic errors, resulting in the participle for "love", "santong ", "Three lanes", "Yantai", etc.; (2) deactivate meaningless words in the text (this step needs to be selected according to the results of the trial run), such as "is from", "open", "between", etc.; (3) Filtering out characters other than Chinese to prevent omissions in the previous manual judgment; (4) Segmentation of comment data.

### Methods

**Figure 3:**
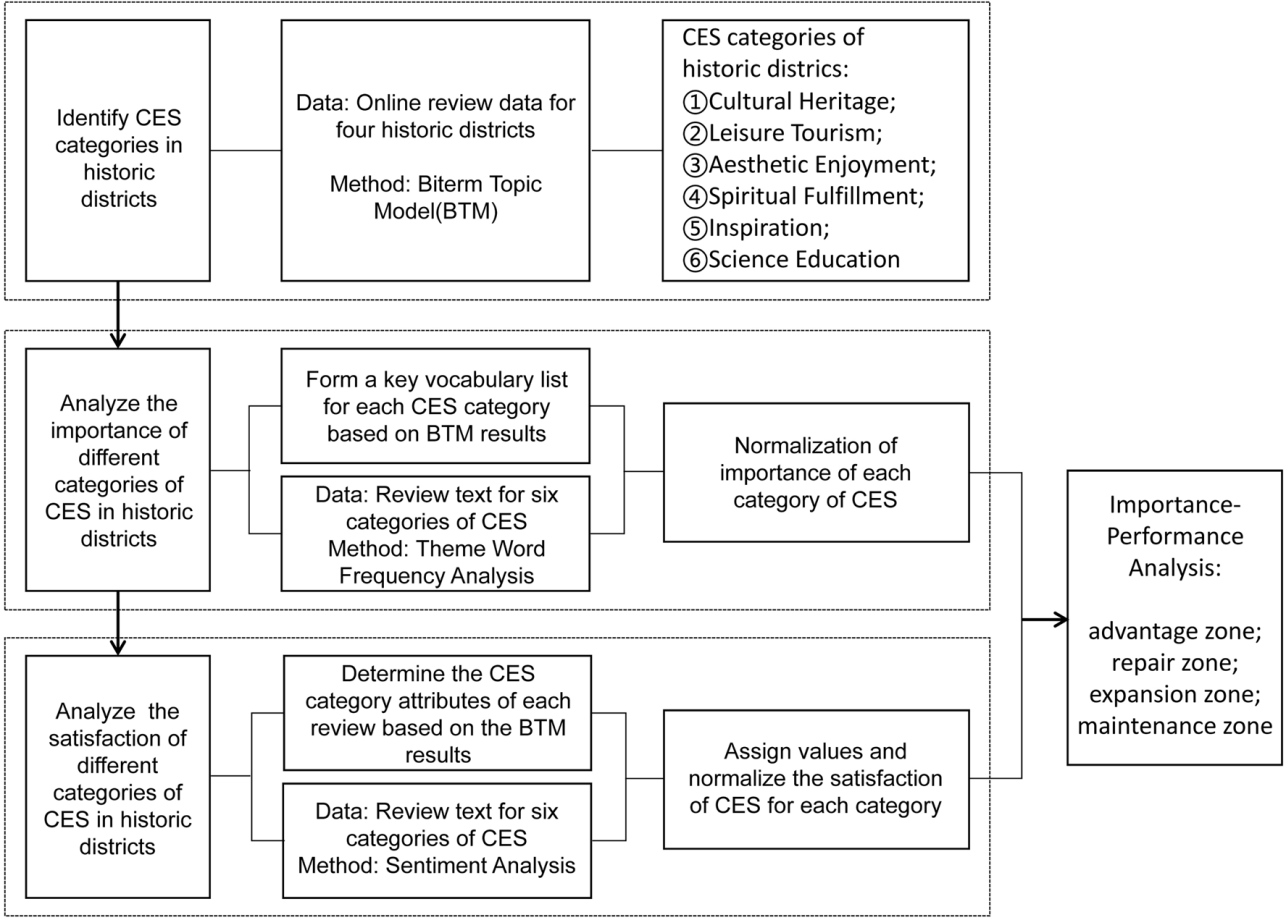
Research framework.

This study first uses the BTM method to summarize and classify the themes of CES in historical districts. At the same time, through the statistics of word frequency, the importance of six CES categories in four historical districts is calculated. Then, sentiment words are extracted using sentiment analysis methods, and sentiment values are calculated for the text based on the calculation program, in order to determine the sentiment tendency of the text based on the sentiment values. And assign values to various emotional outcomes to calculate the satisfaction values of each CES category in the four historical blocks. Finally, based on the assignment of importance and satisfaction, calculate the IPA analysis results. The research framework is shown in Fig. 3.

### Biterm topic model

**Figure 4:**
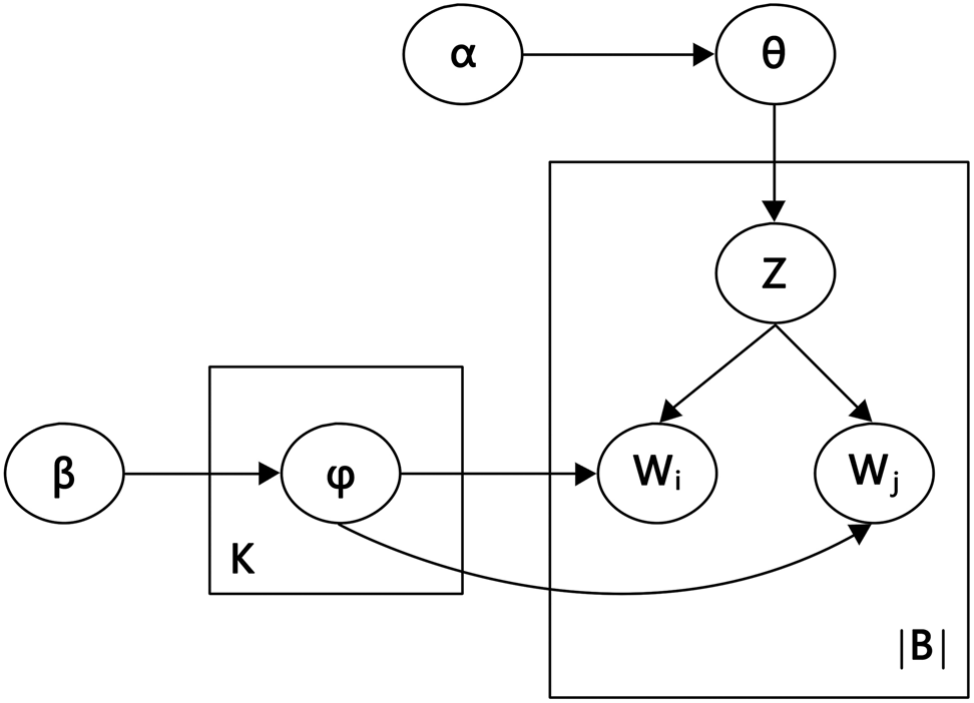
BTM principle process. Where α and β are Dirichlet prior parameters, θ is the probability parameter of a word pair's potential topic in the linguistic corpus, Z is the potential topic in the linguistic corpus, and φ is the probability of the word pair appearing in the topic. The language corpus contains K topics and |B| word pairs, and words W i and W j form word pairs [Wi, Wj].

Bierm topic model (BTM) is an algorithm that reveals topics by modeling global word co-occurrence. The advantage of BTM over other topic models is that it models word co-occurrence in a very explicit way and uses word co-occurrence models aggregated from a corpus for topic discovery^35^. Word pairs are unordered binary groups that appear simultaneously in the text, and modeling with word pairs can alleviate the problem of relatively sparse co-occurrence of individual words in the short text, thus effectively reducing the problem of information loss caused by the segmentation of the short text^51^. The modeling process of BTM consists of four main steps: (1) constructing a linguistic corpus based on short textual data sources; (2) training the model using a word pair corpus; (3) adjusting the computational parameters of the model; (4) generating topic distributions. Its specific process is shown in Fig. 4.

BTM usually measures the quality of the themes found in terms of Coherence or Perplexity. Coherence refers to the frequency of simultaneous occurrences of words within each theme, the more frequent the occurrences, the more coherent the theme^52^. Perplexity represents the explanatory power of the model, and the smaller the value, the better the model explains the text. According to this principle, this study innovatively introduces the calculation of topic validity (TV) to determine the optimal number of topics. The calculation formula is as follows:

$$TV = TP*TC$$

where TV stands for theme validity, TP is theme perplexity, and TC is theme coherence. The closer the TV value is to zero, the greater the number of themes, the better the model's explanatory ability, and the farther it is from zero, the worse the model's explanatory ability.

**Figure 5:**
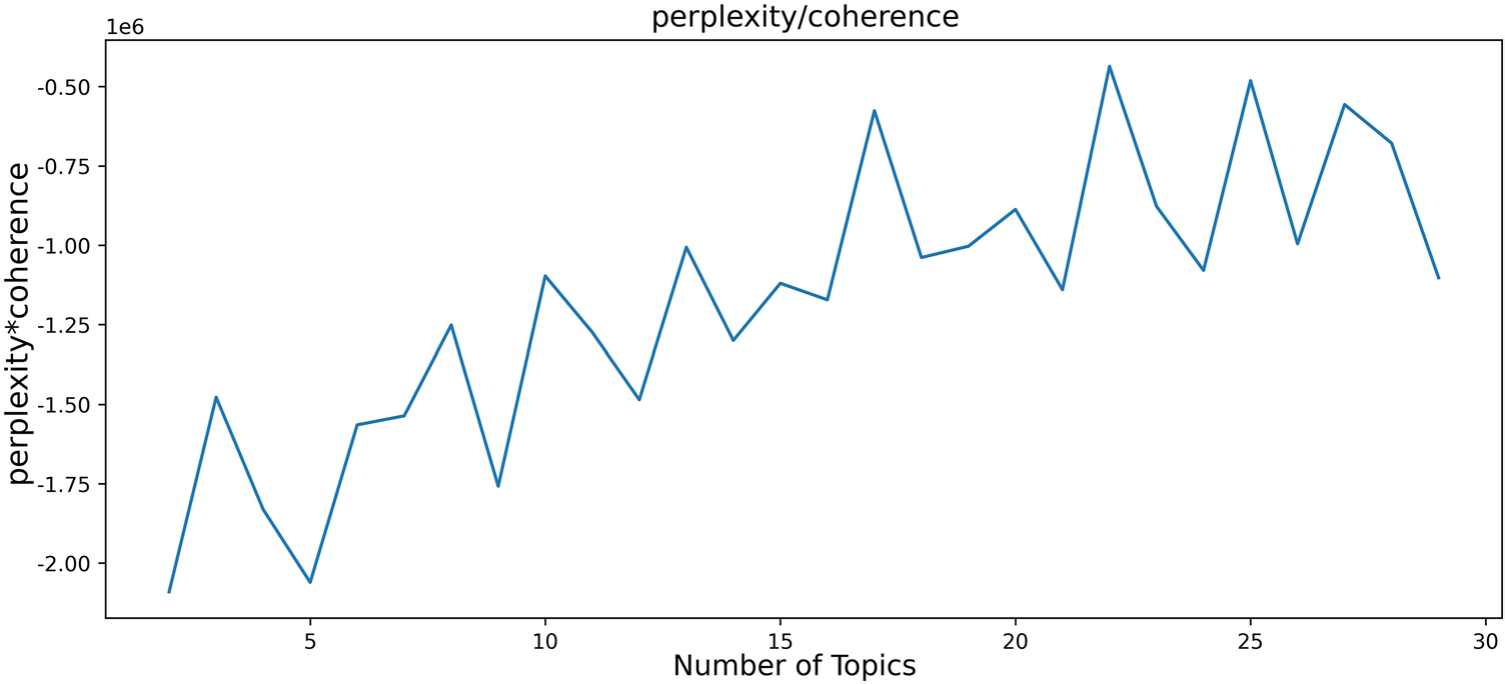
Topic validity values under different number of topics.

When topic modeling the 14,681 comments obtained from preprocessing, the value range of the number of topics K is set to be 2–30, and the interval is 1 to calculate the topic validity scores under different numbers of topics. As shown in Fig. 5, when the number of topics is 22, the topic validity is close to 0, which is the optimal value, so 22 is selected as the number of topics in this study. Finally, the parameters of the BTM were set as follows: number of topics K = 22, α = 50/K, β = 0.01, and 5000 iterations of learning. Two documents can be obtained after running the BTM, which are document-topic distribution and topic-word distribution. The topic-document distribution is the output of the probability of occurrence of each topic in each comment data, with a larger value indicating a higher likelihood of occurrence under that topic. Topic-word distribution, on the other hand, is the probability of each word appearing in different topics, the larger the value indicates that the higher the probability of appearing in the topic, then the topic can be better characterized.

### Sentiment analysis

Sentiment analysis (SA), also known as opinion analysis or opinion mining, is an ongoing field of research in the field of text mining. It is a computational study of exploring people's opinions, attitudes, and emotions towards an entity, that is, opinions, emotions about text and computational processing of subjectivity^53^. With the goal of finding opinions, identifying the sentiments they express, and then categorizing them^54^. At present, the academic field is rich in technical research on SA algorithms, and this study adopts ROST EA1.9.04 to assist in the processing of the processed data. ROST EA1.9.0 is a software developed by Prof. Shen Yang's team at Wuhan University which is specifically used for sentiment analysis. After extracting sentiment words from the text based on the Sentiment Dictionary, the software will calculate the sentiment value of the text based on the calculation procedure and the defined grammatical rules for the text. After extracting the emotional words from the text based on the emotion dictionary, the software calculates the emotion value of the text according to the calculation program and the defined grammar rules, so as to judge the emotion tendency of the text according to the positive and negative emotion values, and classify the emotion into three kinds of positive, neutral and negative emotions^55^. The scoring criteria are: affective tendencies less than 5 as negative emotions, 5 as neutral emotions, and more than 5 as positive emotions^56^.

### Importance-performance analysis

**Figure 6:**
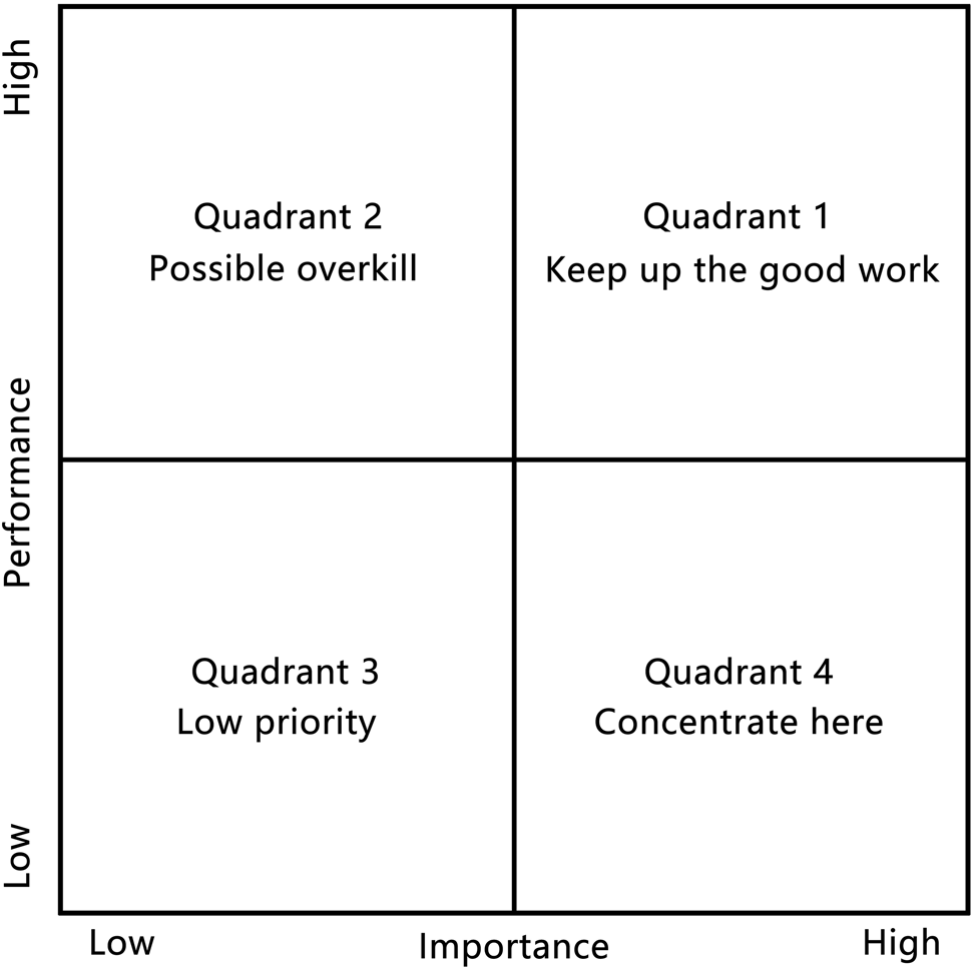
The standard IPA chart (Martilla and James, 1977).

Importance-Performance Analysis (IPA) is a sociological research methodology proposed by Martilla & James^57^. Since its introduction, IPA analysis has been widely used in public satisfaction research, mainly through the analysis of the public's perception of the importance of the object of study and the objective performance of the object of study of the differences in the performance of the object of study, the satisfaction of the object of study evaluation, and based on the results of the optimization of enhancement strategies. As shown in Fig. 6, the core of the IPA analysis is based on the findings of the construction of the importance of the average and the satisfaction value IPA chart, based on the importance of each specific indicator and the average value of satisfaction, were located in the four quadrants of the chart, the first quadrant for the advantage zone; the second quadrant for the repair zone; the third quadrant for the expansion zone; the fourth quadrant for the maintenance zone^58^. Considering the importance and satisfaction of the research object, it is conducive to the rational interpretation of the evaluation indexes and then put forward more reasonable and more targeted optimization and enhancement strategies^59^.

## Analysis and results

### Topic discovery results

#### Topic word frequency statistics

**Figure 7:**
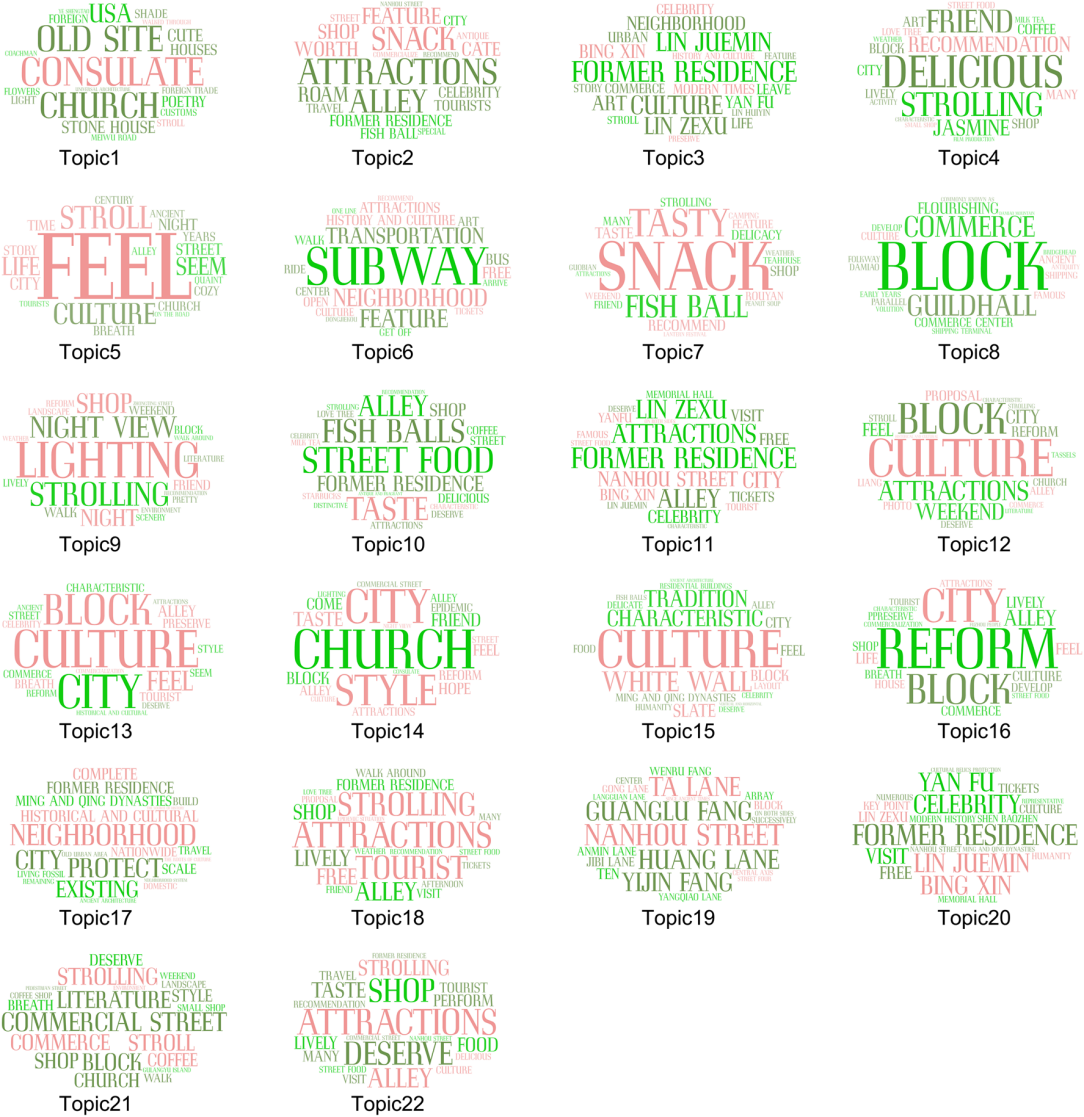
Topic word cloud map.

Two documents and one graph can be obtained after running the BTM, which are the document-topic distribution, topic-word distribution, and topic-word cloud graph. Topic-document distribution is the output of the probability of each topic appearing in each comment data, the larger the value means the higher the possibility of appearing in that topic, and subsequently this study will analyze the sentiment of the comment data under different topics according to this part. Topic-word distribution refers to the probability of occurrence of each word in different topics, the larger the value indicates that the higher the probability of occurrence in the topic, then it can better describe the characteristics of the topic. The topic word cloud is shown in Fig. 5, 22 topics are displayed in the form of a word cloud, the words with the top 20 probability of occurrence are displayed, while the size of each word indicates the probability of occurrence of the word in the theme, as shown in Fig. 7:

### CES categories of historic districts

Due to geographical and cultural differences, the classification of CES indicators in different studies varies somewhat. However, the MEA report's categorization of CES is by far the most widely used(MA,2005), and the main indicators include (1) spirituality and religion; (2) recreation and ecotourism; (3) aesthetics; (4) Inspiration; (5) sense of place; (6) cultural heritage; and (7) education. Based on this framework, some scholars have also proposed their research frameworks, and in 2007 Boyd et al. categorized ecosystem services based on a variety of benefit correlations, including services such as spiritual enjoyment, travel Inspiration, recreation, and entertainment^60^. Emphasizing the relationship between ecological structure/function and human needs related to cultural values, Daniel 2012 elaborates on cultural services from four perspectives: landscape aesthetics, cultural heritage, recreational tourism, and spiritual and religious significance^61^. In 2013 Crossman subdivided existing research on cultural and open space service mapping of ecosystems into five indicators: aesthetics, cognitive development, recreation, art and design Inspiration, and spiritual experience^62^. The Regional Assessment of Biodiversity and Ecosystem Services in the Asia–Pacific Region, published in 2018 at the Intergovernmental Science-Policy Platform on Biodiversity and Ecosystem Services (IPBES), categorizes nature's intangible contributions to humans into four categories: learning and Inspiration, physical and mental experience, support for spirituality, and preserving the right of humans to make good life choices.

This study is based on the content of indicator classification in MEA and related references, and combined with the distribution data of topic words calculated by the BTM, while three expert professors, five PhDs, and three masters were invited to make a preliminary classification of historic district CES^63^. The 22 topics derived from the analysis were matched to the relevant categories of the CES of the historic districts to construct the CES classification system of the historic districts, and the results can be used as the basis for the classification of the CES of the historic districts, as shown in Table 1.

**Table 1:** CES categories of historic districts. Source: Researcher, 2024.

CES categories	Description	Representative words	Include topics
Cultural Heritage	Provide culturally valuable places for people to understand the local culture	Celebrity Former Residence, Guanglu Lane, Huang Alley, Memorial Hall, Guild Hall, Historic Building, Cultural Relics Protection	Topic 8, 11, 15, 19, 20
Leisure Tourism	Provide opportunities for recreation and entertainment, allowing people to relax both physically and mentally	Attractions, Strolling, tourism, Street Food, transportation, Lively, Weekend, Tourist, Shop, Camp	Topic 2, 6, 7, 10, 12, 18, 22
Aesthetic Enjoyment	Provide visual enjoyment and make people feel beautiful	Stone house, Poetry, Cuteness, Flowers, Jasmine, Love Tree, Literature, Scenery, Night View	Topic 1, 4, 9, 21
Spiritual Fulfillment	Provide ideological enlightenment, allowing people to achieve spiritual satisfaction and religious beliefs	Church, Feeling, Feeling, Life, Comfort, Recommendation, Hope	Topic 5, 13, 14
Inspiration	Provide rich sources of Inspiration for art, folk myths, national symbols, architecture, and more	Stories, Celebrities, Culture, Retention, Transformation, Uniqueness, Preservation	Topic 3, 16
Science Education	Provide educational opportunities for individuals to acquire knowledge	Protection, Ming and Qing Dynasties, Extant, Living Fossils, Neighborhood System	Topic 17

The number of comments varied by historic district, with the popular, large, historic, and earliest updated Three Lanes and Seven Alleys historic district receiving the highest number of comments at 8036, while the smaller, most recently opened Liangcuo historic district, which is the smallest and most recently opened, received the fewest total comments with only 732. There are also differences in the cultural service categories of different urban historic districts, and the Three Lanes and Seven Alleys historic district, which has been rated as a 5A-level tourist attraction, to a certain extent undertakes the functions of the city's main cultural services. The proportion of the average CES score of each of the four historic districts in Fuzhou is shown in Table 2. Among the different CES types, the type of Leisure Tourism is the most easily perceived by the public, with a share of 46.1%, and Liangcuo historic district among the four historic Districts has the highest value of Leisure Tourism, with a share of 60.3%. Aesthetic Enjoyment comes next, accounting for 26.5%, with Yantai Hill historic district having the highest Aesthetic Enjoyment value of 40.8%. The Science Education category is the least likely to be perceived by the audience, accounting for only 2.1% of the total. As an urban historic district, its most prominent value of Cultural Heritage is ranked third in the public perception with only 10.1%, which is similar to the value of Spiritual Fulfillment (9.9%).

**Table 2:** Percentage of CES value by historic district. Source: Researcher, 2024.

Name of historic district	Number of comments	Cultural heritage	Leisure tourism	Aesthetic enjoyment	Spiritual fulfillment	Inspiration	Science education
Three Lanes and Seven Alleys	8036	0.171	0.479	0.138	0.095	0.057	0.060
Shangxiahang	2671	0.137	0.385	0.284	0.124	0.059	0.011
Yantai Hill	3242	0.048	0.377	0.408	0.109	0.054	0.005
Liangcuo	732	0.048	0.603	0.229	0.069	0.041	0.009

### Sentiment analysis results

The statistical results of sentiment analysis are shown in Table 3, which shows that the public's overall satisfaction with the Fuzhou historic district is high. Among the 14,681 comments, there are 11,834 positive emotions, accounting for 80.61%; 78 neutral emotions, accounting for only 0.53%; and 2769 negative emotions, accounting for 18.86%. Thus, it can be seen that the overall satisfaction of tourists with the historic district is high.

**Table 3:** Summary table of emotional attitude statistics for historic districts web text data. Source: Researcher, 2024.

Variable	Quantity/piece	Proportion
Positive emotion	11,834	80.61%
Neutral emotion	78	0.53%
Negative emotion	2769	18.86%
Segmented statistical results of positive emotions		
General (5,15]	2539	17.29%
Moderate (15,25]	2482	16.91%
High (25, + ∞)	6813	46.41%
Segmented statistical results of negative emotions		
General (− 15,5]	595	4.05%
Moderate (− 25,− 15]	223	1.52%
High (− ∞,− 25)	113	0.77%

Through the statistical results of sentiment distribution of the topic sentences assigned to each of the six categories of CES cultural services, it was found that, as shown in Table 4, the category of popular Science Education had the highest positive sentiment of 91.14%, while Inspiration had the lowest satisfaction level of 75.1 and the highest negative sentiment of 24.7%.

**Table 4:** Historic district CES emotional attitude statistics. Source: Researcher, 2024.

Variable	Cultural heritage	Leisure tourism	Aesthetic enjoyment	Spiritual fulfillment	Inspiration	Science education
Positive emotion	82.47%	78.87%	83.31%	76.68%	75.10%	91.14%
Neutral emotion	1.12%	0.44%	0.40%	0.81%	0.20%	0.68%
Negative emotion	16.41%	20.69%	16.29%	22.51%	24.70%	8.18%

After further word frequency extraction of the results of the six dimensions of negative sentiment, this study found that the public's negative sentiment towards ecosystem cultural services in historic districts is concentrated in the areas of "commercialization", "similarity", "uniformity", "destruction", "touristy", "disappointment", "fees", and "uniqueness". uniformity", "destruction", "sightseeing", "disappointment", "fees", "crowded", "boring" etc. By extracting keywords of positive emotions, it can be found that the high-frequency By extracting the keywords of positive emotions, it can be found that high-frequency positive emotions are "tourist place", "trend", "punch card", "comfortable", "perfect", "comfortable", "perfect", "complete", "entertainment", "convenient", " worthwhile", "leisure" and other words.

### IPA analysis results

**Figure 8:**
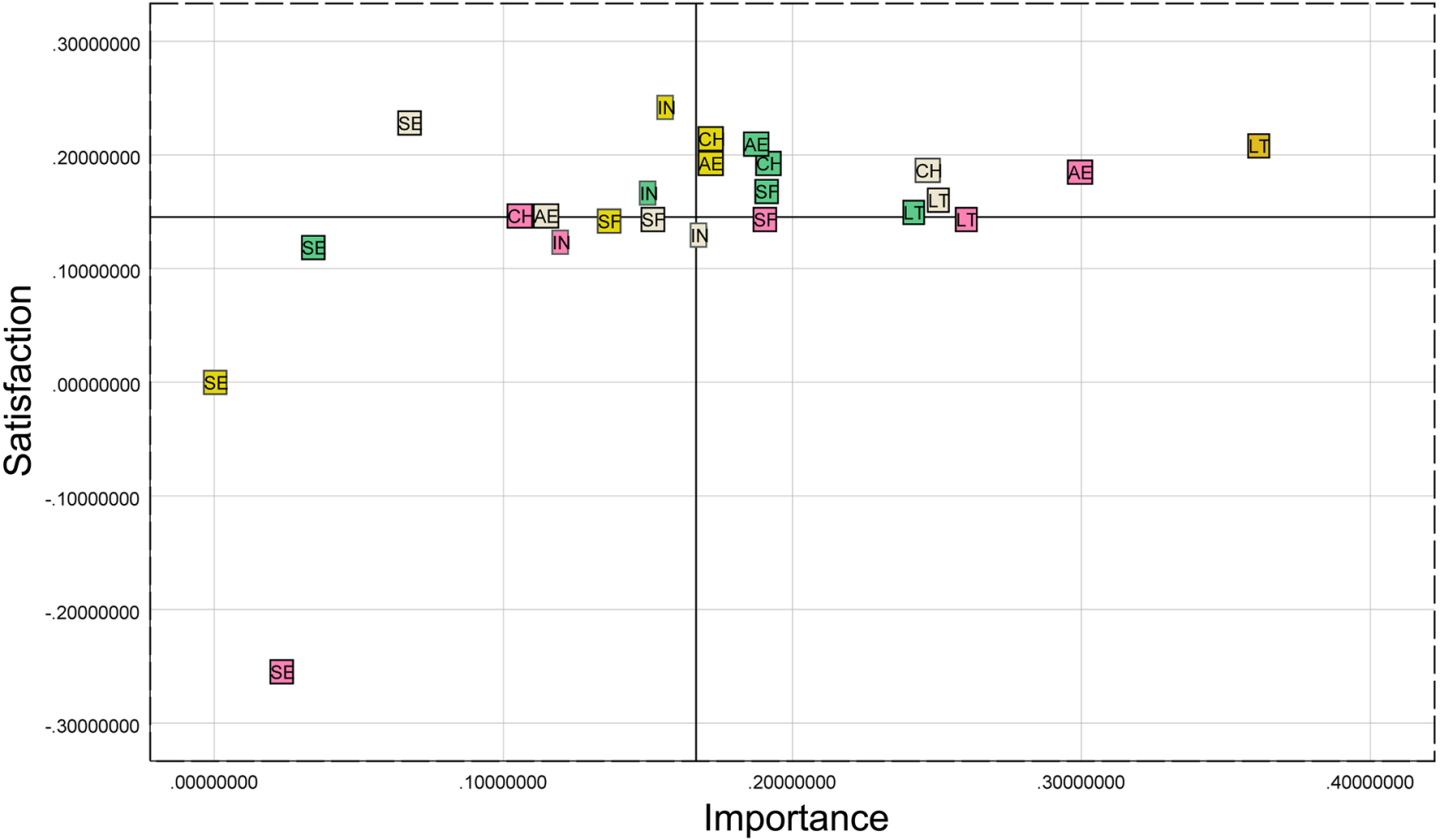
IPA quadrant map of historic districts in Fuzhou.

The 22 topic word frequencies from the previous topic discovery section were used for categorized word frequency statistics to derive the word frequency ranking of the four historic districts in the six CES categories, and the word frequency percentage was used as the degree of importance of each category. At the same time, the sentiment analysis of each historic district according to the six categories of CES was conducted, the sentiment results of each category were assigned, and the resulting sentiment scores were used as the satisfaction level of each historic district in the CES categories. Scatter plots were drawn through SPSS25.0 software, and the average value of the indicators at all levels was taken as the dividing line of the horizontal and vertical coordinates, and the results of the IPA analysis obtained are shown in Fig. 8.

The six indicators for each of the four historic districts fall into four different quadrants. The gray squares represent the six CES indicators of the Three Lanes and Seven Alleys historic district; the green squares represent the six indicators of the Shangxiahang historic district; the pink squares represent Yantai Hill historic district; and finally, the yellow squares represent Liangcuo historic district. The results of IPA analysis show that: (1) the Cultural Heritage and Leisure Tourism indicators of Three Lanes and Seven Alleys fall into the dominant zone, which indicates that Three Lanes and Seven Alleys has relatively good performance in these two aspects of the CES, and can provide the public with a certain degree of Cultural Heritage and Leisure Tourism and Science Education indicator falls into the repair area, with low importance and high satisfaction, indicating that although Science Education is not the most concerned part of the public, the existing Science Education function of Three Lanes and Seven Alleys is still the most important part of the public's attention. Science Education is not the most concerned part of the public, but the existing Science Education function of Three Lanes and Seven Alleys has still gained high satisfaction from tourists. (2) Aesthetic Enjoyment, Cultural Heritage, and Spiritual Fulfillment of the historic districts of Shangxiahang all fall into the first quadrant, but Inspiration fells into the second quadrant, and Science Education fells into the third quadrant, indicating that the public's overall satisfaction with the CES services provided by the historic districts of Shangxiahang is relatively high, but that improvements can be made to the part of Science Education to improve the overall satisfaction with the historic districts of Shangxiahang. However, the Science Education section can be improved to increase the overall satisfaction of the Shangxiahang historic district. (3) The Yantai Hill historic district, which has been open for a relatively short period, has a high level of satisfaction and importance in the Aesthetic Enjoyment section but also needs to be improved in the Science Education Sect. (4) Liangcuo historic district, which is relatively far away, has a better performance in Leisure Tourism, but the construction of Inspiration can be increased appropriately.

## Discussions

In the current study, most of the researchers used questionnaires or geographic information data as the base data for calculations to conduct quantitative research on CES^64,65^. This study innovatively uses online text data as the basis, models the corpus of CES using BTM, and conducts matching research with CES based on topic word distribution, establishing the relationship between online review data and cultural service types. Meanwhile, combining sentiment analysis and IPA analysis provides new possibilities for the quantification of CES in urban historic districts.

In the Cultural Heritage dimension, although all four historic districts do not have a high level of perception, the Three Lanes and Seven Alleys historic district, as a 5A-level tourist attraction in Fuzhou, has a relatively high level of public perception in this regard, reaching 17.1%; the Shangxianghang historic district is second only to the Three Lanes and Seven Alleys historic district, with a level of 13.7%; the Yantai Hill historic district and the Liangcuo historic district, as historic districts developed late, have a relatively low level of public perception of their Yantai Hill historic district and Liangcuo historic district, which were developed later, have a lower perception of their Cultural Heritage dimension, both at only 4.8%. In the dimension of Leisure Tourism, all four historic districts have the highest level of perception among the six categories, with 47.9% for the Three Lanes and Seven Alleys historic district, 38.5% for the Shangxiahang historic district, 37.7% for the Yantai Hill historic district, and 60.3% for the Liangcuo historic district. In the module of Spiritual Fulfillment, all four historic districts do not have a high degree of perception, and the Three Lanes and Seven Alleys, which is the cultural card of Fuzhou, is only 9.5%, while Shangxiahang historic district has the highest degree of perception in this part, 12.4%, and Liangcuo historic district has the lowest degree of perception of spiritual satisfaction, only 6.9%. In the section of Aesthetic Enjoyment, the Yantai Hill historic district, as a Tourist Attraction that has not been open for a long time but is popular with young people, received the highest value of 40.8%, indicating that the Aesthetic Enjoyment section of Yantai Hill is the easiest to perceive, while the Aesthetic Enjoyment section of Three Lanes and Seven Alleys is not easy to be perceived by the public, with a perception level of 13.8%, which is the lowest value under this category.

In previous studies, although Leisure Tourism is regarded as the most important CES function^28^, as it is most often mentioned and evaluated, it is mostly seen in large-scale areas or areas such as parks where leisure activities are dominant^66^. As a relatively small scale, the historic district with the most prominent cultural characteristics should not focus its enhancement on the development of leisure and tourism but should concentrate its main value on the two parts of cultural heritage and Spiritual Fulfillment, to achieve the effect of point activation of urban culture and revitalization of urban culture.

In this part of the sentiment analysis, the public's evaluation of the positive sentiment of the perceived Cultural Heritage of Fuzhou's historic districts is more than the negative sentiment, with 80.61% positive sentiment and 18.86% of negative sentiment. Online reviews express the public's emotions and feelings about the historic districts visited, and grasping the negative comments therein is meaningful for enhancing the value of the cultural services of the neighborhood ecosystems, which can be targeted to suggest improvements to the relevant content. By analyzing the negative emotional comments on the six dimensions of CES, the following are summarized: (1) the negative comments on the cultural heritage dimension mainly mentioned that the development of the historic districts was too rigid, serious damage, serious homogenization, and lack of historical and cultural heritage, etc.; (2) the Leisure Tourism dimension has the highest degree of perception, so it also has the most negative comments, which focused on the fact that it is easy to get lost, the taste of the snacks are not cheap, the architecture is too commercialized, and the private houses are not open to the public. too many stores selling things, much the same as other cities' historic districts, the weather is too hot, too few parking spaces, etc.; (3) the negative sentiment of the Aesthetic Enjoyment dimension mainly mentioned: too many tourists, only walking around, not the original ancient flavor, no sense of historical gravity, the scenery has no characteristics, etc.; (4) the negative comments of the Spiritual Fulfillment dimension are mainly: Not feeling the cultural atmosphere, missing the old ancient neighborhoods (regret), now turned into a food street (sad), less flavor of life, artificial restoration of the thousand and one without characteristics. (5) Inspiration and Science Education are perceived to be lower, and their negative comments mainly include less sense of historical vicissitudes, commercial destruction of the environment, no sense of ancient meaning in the ancient architectural complexes, hope to preserve the original ancient architecture, big difference between the actual attractions and the pictures on the Internet, and regret for not having guided tours to explain the contents, and so on.

Finally, this study conducts an IPA analysis of public perception of CES in Fuzhou's historic districts through the results of topic discovery and sentiment analysis. In this part, most of the twenty-four indicators are located in the first quadrant of the quadrant map, and some of the indicators are located in quadrants 2 and 3, indicating that the overall public satisfaction with the CES of Fuzhou's historic districts is good. Based on the results of the IPA analysis, the CES functions of the four historic districts are proposed to be improved.

In summary, this study studied six CES perception dimensions in four historic districts in Fuzhou to understand the types of cultural services that historic districts can provide and the public's perception of different types of cultural services. Based on the analysis results, targeted improvement suggestions are put forward to meet the public's spiritual needs for traveling to historic districts, improve the overall perceived satisfaction of the neighborhood, thereby enhancing cultural identity, promoting sustainable renewal, and improving public well-being.

## Conclusions and future study

This study uses online review data as the data source, combined with the BTM, to clarify the types of CES that historic districts can provide, and conducts sentiment analysis and IPA analysis on various types of CES, thereby proposing improvement suggestions for the development of Fuzhou historic districts.

Through the calculation of the BTM, CES in Fuzhou's historic districts is divided into six categories, namely cultural heritage, leisure tourism, aesthetic enjoyment, spiritual fulfillment, inspiration, and science education. Among them, leisure tourism and aesthetic enjoyment account for the highest proportion, indicating that the development of leisure tourism in Fuzhou's historic districts is relatively complete. As for the area with the most prominent cultural heritage in the city, the public's perception of its cultural heritage accounts for only 10.1%. It can be seen that the public does not have a particularly prominent perception of the cultural heritage dimension. Therefore, when updating historic districts, more consideration should be given to how to enhance the public's perception of the cultural heritage dimensions of urban historic districts, thereby increasing the city's sense of identity. At the same time, this study conducted sentiment analysis and IPA analysis on classified online texts and found that the public is generally satisfied with the Fuzhou historic district. In response to negative evaluations, the following improvement suggestions are put forward: (1) Regarding the Three Lanes and Seven Alleys Historic District , it is necessary to focus on improving its spiritual satisfaction and aesthetic enjoyment, and strengthening the function of popular science education; (2) The focus of Shangxiahang Historic district should be on the two parts of inspiration and science education; (3) The historic district of Yantai Hill Historic District needs to enhance the cultural heritage part; (4) Liangcuo Historic District needs to focus on strengthening spiritual fulfillment and inspiration.

This study focuses on urban historic districts with high demand for cultural services, employing the CES theory to conduct research. The research findings possess theoretical validity and offer the following research values: (1) Providing a new perspective on historic district research, injecting relevant CES theory into historic district renewal can not only effectively enhance the cultural service functions of historic districts but also contribute to increasing public awareness of ecosystem and cultural heritage conservation. (2) This study enhances the importance of public perception in historic district development. By exploring the CES in historic district , it meets tourists' spiritual needs, thereby enhancing human well-being. (3) Online review data express the public's emotions and feelings about the historic districts they visit. Grasping the evaluation content is meaningful for enhancing the value of CES, and targeted improvement suggestions can be made for relevant content.

However, since this study uses Internet text data mining to collect public perception data, whether the source of tourist review data obtained through Dianping.com is too single. Moreover, it is not difficult to find from the collected data results that the review data of the four historic districts have a large difference in the number of reviews due to various reasons, and there is an imbalance. Therefore, in the future, multi-source data collection methods will be considered for data collection to make the data sources more diverse, and research will be carried out using a combination of quantitative and qualitative methods to make the data coverage wider and improve the accuracy of the results.

## Data Availability

The datasets used and analyzed during the current study are available from the corresponding author on reasonable request.
